# Supplementary material for: Fitness Landscape for Antibodies 2: Benchmarking Reveals That Protein AI Models Cannot Yet Consistently Predict Developability Properties
Source: bioRxiv. 2025 Dec 27:2025.12.27.696706. Preprint. [Version 1] doi: 10.64898/2025.12.27.696706 (PMC12767642; doi:10.64898/2025.12.27.696706)
Supplement: 1 [file NIHPP2025.12.27.696706V1-supplement-1.pdf]

bioRxiv preprint doi: https://doi.org/10.64898/2025.12.27.696706; this version posted December 27, 2025. The copyright holder for this preprint (which was not certified by peer review) is the author/funder, who has granted bioRxiv a license to display the preprint in perpetuity. It is made available under aCC-BY 4.0 International license.

10 Supplementary

Table of contents

10.0 Data availability **25**

10.1 Data composition
Table S1: Unique antibody sequence-fitness label pairs from antibody datasets 25
Figure S1: Assays present in each developability category 26

10.2 Descriptions of developability properties **26**
Thermostability 26
Expression 26
Aggregation 26
Binding affinity 27
Pharmacokinetics 27
Polyreactivity 27
Immunogenicity 27

10.3 Protein AI model descriptions **27**
Table S2: Model descriptions 28
Decoder-only models 29
Encoder-only models 29
Inverse folding models 29
Structure prediction models 29
Physics-based models 29
Germline model 30

10.4 Zero-shot results **30**
Table S3: Datasets that pass the 5-significant-model cutoff 30
Table S4: Zero-shot performance summary across all models 33
Table S5: Zero-shot performance summary for each individual model 33
Table S6: Zero-shot performance across architectures 34
Table S7: Zero-shot performance across sequence-only and structure-informed models . 34
Table S8: Zero-shot performance across general protein and Ab-specific AI models . . . 35
Figure S2: Zero-shot prediction compared to parameter size 35
Figure S3: Distribution of zero-shot prediction correlations with germline 36
Table S9: Zero-shot correlation with germline signal 37
Table S10: Zero-shot correlation to germline across sequence or structure models 37
Figure S4: Zero-shot prediction germline bias compared to parameter size 38
Figure S5: Zero-shot prediction performance with varying directions of germline 39

bioRxiv preprint doi: https://doi.org/10.64898/2025.12.27.696706; this version posted December 27, 2025. The copyright holder for this preprint (which was not certified by peer review) is the author/funder, who has granted bioRxiv a license to display the preprint in perpetuity. It is made available under aCC-BY 4.0 International license.

Partial correlation coefficient calculation 39
Table S11: Partial correlation datasets 39
Table S12: Partial correlations adjusted for germline signal 40

10.5 Few-shot results **41**
Table S13: Datasets that pass the 5-significant-model cutoff 41
Figure S6: Count of significant correlations per few-shot model 43
Table S14: Few-shot performance summary across all models 44
Table S15: Few-shot performance summary for each individual model 44
Table S16: Few-shot performance across architectures 44
Table S17: Few-shot performance across sequence- and structure-informed models 44
Table S18: Few-shot performance across general protein and Ab-specific AI models . . . 45
Figure S7: Distribution of few-shot prediction correlations with germline 46
Table S19: Few-shot correlation with germline signal 47
Figure S8: Distribution of few-shot prediction correlations with germline 47

bioRxiv preprint doi: https://doi.org/10.64898/2025.12.27.696706; this version posted December 27, 2025. The copyright holder for this preprint (which was not certified by peer review) is the author/funder, who has granted bioRxiv a license to display the preprint in perpetuity. It is made available under aCC-BY 4.0 International license.

10.0 Data availability

Data, scripts, and analyses are available at https://github.com/Graylab/FLAb/releases/tag/v1.0.0.

The datasets collected and used in our analyses are covered by a diverse set of licenses, and we only share datasets in our GitHub that allow redistribution (Attribution 4.0 International, Attribution-NonCommercial 4.0 International, Attribution-NonCommercial-NoDerivatives 4.0 International, MIT, Clear BSD). The datasets not redistributed in the FLAb2 GitHub are from Ginkgo Datapoints[12] (https://huggingface.co/datasets/ginkgo-datapoints/GDPa1) and NaturalAntibody[35,79] (https://naturalantibody.com/ab-design/, https://naturalantibody.com/therapeutic-antibody-database/).

10.1 Data composition

Table S1: Number of unique antibody sequence-fitness label pairs from antibody datasets. The data provided by Garbinski et al. (2023) is not associated with a publication.

Study	Tm.	Exp.	Agg.	Bind.	PK.	Poly.	Imm.
Jain et al. (2017)[39]	137	137	685			685	
Jain et al. (2023)[80]	137		685		262	1707	
Jetha et al. (2019)[81]			97				
Shanehsazzadeh et al. (2023)[31]	39		65	461	39	28	
Jain et al. (2024)[10]	43		301	33	129	86	
Arsiwala et al. (2025)[12]	682	324	1235			488	
Kraft et al. (2019)[82]			128				
Phillips et al. (2021)[83]				67033			
Engelhart et al. (2022)[84]				352140			
Shanker et al. (2024)[7]				223		39	
Shanehsazzadeh et al. (2024)[85]				321			
Garbinski et al. (2023)	86	94		81			
Hie et al. (2023)[6]	37			146			
Hutchinson et al. (2023)[52]	258			362			
Koenig et al. (2017)[86]		4275		4275			
Makowksi et al. (2022)[87]				126			
Rawat et al. (2022)[88]				569			
Rosace et al. (2023)[89]	19			19		38	
Warszawski et al. (2019)[90]				2048			
Zimmerman et al. (2020)[91]				21			
Adams et al. (2017)[92]				11067			
Peterson et al. (2024)[93]				2111			
Kirby et al. (2024)[78]				2276			
Erasmus et al. (2024)[94]				142			
Tsuruta et al. (2024)				573891			
Krawczyk et al. (2025)[35]				1328			
Kothiwal et al. (2025)[95]				709			
Marks et al. (2021)[96]							217
Natural Antibody (2025)[97]							4855
Sulea et al. (2019)[98]	33						

bioRxiv preprint doi: https://doi.org/10.64898/2025.12.27.696706; this version posted December 27, 2025. The copyright holder for this preprint (which was not certified by peer review) is the author/funder, who has granted bioRxiv a license to display the preprint in perpetuity. It is made available under aCC-BY 4.0 International license.

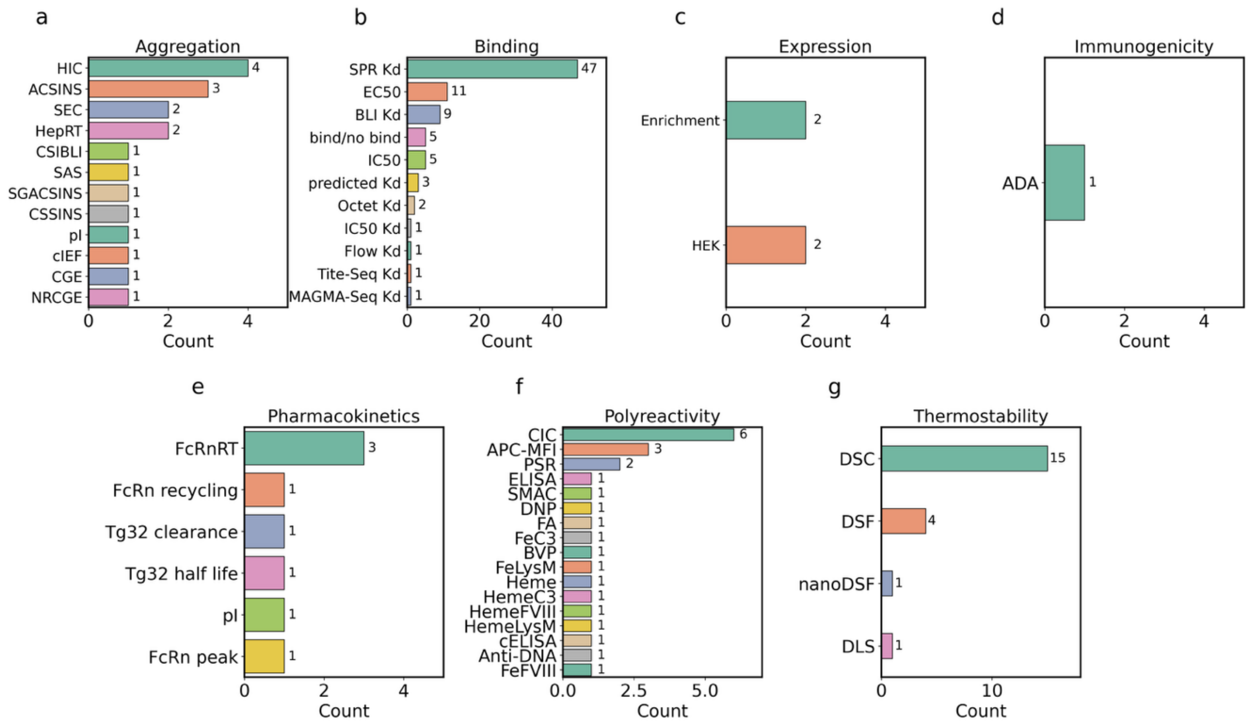

Figure S1: Assays present in each developability category. Each developability property can be measured with a variety of different assays, and we present the counts of each collected assay.

10.2 Descriptions of developability properties

Thermostability ensures an antibody will maintain its structure and function when exposed to heat, particularly during manufacturing, storage, and administration. Antibodies with high thermostability are more likely to remain potent over extended periods and under different storage conditions. Differential scanning fluorimetry (DSF) and calorimetry (DSC) is provided for clinical stage and germline antibodies, with some datasets including point mutations of a starting antibody candidate. The datasets range in size - some are a few hundred antibody variants from the same wildtype[6,10,12,31,52,80,89], some are collections of diverse antibodies from other public datasets like NbThermo[99], and some are only 2-3 sequences in dataset size[6].

Expression ensures the production of antibodies in a host cell system, which is necessary to isolate a molecule for further testing and directly affects production yield and cost of manufacturing. Expression data is collected from clinical stage therapeutics[14,39] and rounds of enrichment of a starting antibody candidate[86].

Aggregation refers to the process of individual antibodies coming together to form larger assemblies, or aggregates. Aggregation can be problematic as it leads to reduced therapeutic efficacy and potentially harmful immune responses. Aggregation data is collected for both

bioRxiv preprint doi: https://doi.org/10.64898/2025.12.27.696706; this version posted December 27, 2025. The copyright holder for this preprint (which was not certified by peer review) is the author/funder, who has granted bioRxiv a license to display the preprint in perpetuity. It is made available under aCC-BY 4.0 International license.

therapeutic[12,39,80] and germline[10] antibody datasets, and a breadth of assays for measuring this property (**Fig. S1a**).

Binding affinity ensures the prolonged physical contact during an interaction between an antibody and target antigen to initiate a mechanism of action: Impacting an antigen's ability to block pathways (blocking/antagonist); binding a cell surface receptor to activate some downstream response (agonist); initiation of cellular cytotoxicity, phagocytosis, or apoptosis; or receptor internalization and down modulation[100]. Binding affinity data is collected for therapeutic and germline antibodies, in varying sequence diversity from completely different isotypes to single point deep mutational scans.

Pharmacokinetics refers to how the body of an animal interacts with an administered therapeutic. A complete pharmacokinetic profile encompasses absorption, distribution, metabolism, and excretion. The pharmacokinetic data collected measures the clearance of a drug through mice[10], which provides an indication of the drug's half-life and therapeutic efficacy per dose.

Polyreactivity refers to the promiscuity of antibodies to bind to multiple antigens. Although bispecific and trispecific antibodies do exist[1] and continue to be explored as powerful clinical drug candidates, polyreactivity is generally undesirable as it indicates off-target and non-specific binding. Polyreactivity data is collected for both therapeutic[12,39] and germline[10] antibodies.

Immunogenicity refers to the elicitation of an undesirable immune response after administration of a therapeutic antibody, leading to the generation of anti-drug antibodies (ADAs). ADAs can recognize and neutralize therapeutic antibodies, reducing their efficacy and potentially causing adverse effects. Minimizing immunogenicity is important for therapeutic antibodies to maintain their efficacy and safety. Immunogenicity datasets report the percent of the patient population that exhibited an ADA response after therapeutic administration[79,96]. Each therapeutic drug in these datasets are tested in different patient populations, so the reported ADA response rates are not directly comparable across drugs.

10.3 Protein AI model descriptions

Table S2: Model descriptions. A description of each benchmarked model is provided in terms of its architecture, the model size, the training data, what representations of protein the model has learned, and the output from the model that is used for zero-shot benchmarking.

Model	Architecture	Param size	Train data	Data rep.	Zero-shot val
IgLM[40]	Autoregressive LM	12889600	Antibody	Sequence	Perplexity
ProGen2 Small[41]	Autoregressive LM	151000000	General	Sequence	Perplexity
ProGen2 Medium[41]	Autoregressive LM	764000000	General	Sequence	Perplexity
ProGen2 Base[41]	Autoregressive LM	764000000	General	Sequence	Perplexity
ProGen2 OAS[41]	Autoregressive LM	764000000	Antibody	Sequence	Perplexity

bioRxiv preprint doi: https://doi.org/10.64898/2025.12.27.696706; this version posted December 27, 2025. The copyright holder for this preprint (which was not certified by peer review) is the author/funder, who has granted bioRxiv a license to display the preprint in perpetuity. It is made available under aCC-BY 4.0 International license.

ProGen2 BFD90[41]	Autoregressive LM	2700000000	General	Sequence	Perplexity
ProGen2 Large[41]	Autoregressive LM	2700000000	General	Sequence	Perplexity
ProGen2 XLarge[41]	Autoregressive LM	6400000000	General	Sequence	Perplexity
AntiBERTy[42]	Masked LM	26000000	Antibody	Sequence	Pseudo-ppl
ESM2 8M[43]	Masked LM	8000000	General	Sequence	Pseudo-ppl
ESM2 35M[43]	Masked LM	35000000	General	Sequence	Pseudo-ppl
ESM2 150M[43]	Masked LM	150000000	General	Sequence	Pseudo-ppl
ESM2 650M[43]	Masked LM	650000000	General	Sequence	Pseudo-ppl
ESM2 3B[43]	Masked LM	3000000000	General	Sequence	Pseudo-ppl
ISM 650M[44]	Masked LM	650000000	General	Seq and structure	Pseudo-ppl
ISM 650M[44]	Masked LM	650000000	General	Seq and structure	Pseudo-ppl
ISM 3B[44]	Masked LM	3000000000	General	Seq and structure	Pseudo-ppl
ESM IF[46]	Inverse folding model	142000000	General	Seq and structure	Perplexity
ProteinMPNN[47]	Inverse folding model	1381000	General	Seq and structure	Perplexity
AbMPNN[45]	Inverse folding model	1381000	Antibody	Seq and structure	Perplexity
Chai 1[49]	Structure prediction	3000000000	General	Seq and structure	pLDDT
IgFold[48]	Structure prediction	1600000	Antibody	Seq and structure	pRMSD
BP aromaticity[51]	Biophysics model	NA	NA	Sequence	Unitless
BP charge pH 7.4[51]	Biophysics model	NA	NA	Sequence	Net charge
BP flexibility[51]	Biophysics model	NA	NA	Sequence	Unitless
BP gravy[51]	Biophysics model	NA	NA	Sequence	Hydropathy
BP instability[51]	Biophysics model	NA	NA	Sequence	Unitless
BP pI[51]	Biophysics model	NA	NA	Sequence	pH
BP mol. weight[51]	Biophysics model	NA	NA	Sequence	Daltons
PyRosetta[50]	Biophysics model	NA	NA	Seq and structure	Total energy
LD from GL	Immunological model	NA	NA	Sequence	Unitless

Decoder-only language models

Decoder-only language models have proven to be effective in generating plausible and novel protein sequences. These models are trained using a next-token prediction objective, where the probability of the next amino acid is influenced by the entire preceding sequence. During training, a database of sequences is utilized to predict $p(s_i|s_{<i})$, enhancing the model's ability to generate accurate sequences. We evaluate the zero-shot prediction of therapeutic properties by correlating to the perplexity of each sequence under those models:

$$\mathrm{ppl}(x) = \exp\left(-\frac{1}{n}\sum_{i=1}^{n} \ln p(x_i|\, x_{<i})\right), \qquad (1)$$

where $x = (x_1, x_2, \dots, x_n)$ is a sequence consisting of n tokens.

The decoder models we benchmark are **IgLM** and **ProGen2**. The ProGen2 models come in various sizes, ranging from 151M to 6.4B parameters, pretrained on a mixture of UniRef90 and BFD90 databases. IgLM formulates the design task based on text-infilling using a standard left-to-right decoder (GPT-2), trained on a non-redundant set of 558M antibody sequences obtained from OAS.

bioRxiv preprint doi: https://doi.org/10.64898/2025.12.27.696706; this version posted December 27, 2025. The copyright holder for this preprint (which was not certified by peer review) is the author/funder, who has granted bioRxiv a license to display the preprint in perpetuity. It is made available under aCC-BY 4.0 International license.

Encoder-only language models

Encoder-only language models capture comprehensive information in a continuous abstract representation that can be broadly applied. A subset of residues is randomly chosen and replaced with a special mask token. The model is then trained to predict the identities of these masked residues.

In an encoder-only model, an estimation of perplexity can be obtained by calculating the exponential of the negative pseudo-log-likelihood, or pseudo-perplexity:

$$\text{pseudo ppl}(x) = \exp\left(-\frac{1}{n}\sum_{i=1}^{n} \ln p\left(x_i \middle| x_{\{\backslash i\}}\right)\right), \qquad (2)$$

where $x_{\{-i\}}$ is the set of all residues except x_i.

In this category of models we focus on **AntiBERTy**, a 26M parameter model pretrained on 558M natural antibody sequences from OAS; **ESM-2**, a suite of models ranging in size from 8M to 3B parameters pretrained on 65M unique sequences from UniRef50 and Uniref90; and **ISM**, which includes the 650M and 3B parameter ESM-2 models distilled with structural representations.

Inverse folding models

Generative deep learning architectures that predict protein sequences from structures are known as inverse folding models. For inverse folding models we evaluate the perplexity for each antibody sequence-structure pair:

$$\text{ppl}(x) = \exp\left(-\frac{1}{n}\sum_{i=1}^{n} \ln p\left(x_i \middle| x_{\{\backslash i\}}, \text{structure}\right)\right). \qquad (3)$$

ESM-IF uses an autoregressive encoder-decoder architecture, where the model is tasked with recovering the native sequence of the protein from the coordinates of its backbone atoms. **ProteinMPNN** uses a message-passing neural network with 1.4M parameters that predicts protein sequences using the protein backbone geometry. **AbMPNN** uses the same architecture as ProteinMPNN, with weights obtained from training on antibody PDB structures. Structures of all antibody mutants are predicted with Chai-1 prior to scoring with inverse folding models.

Structure prediction models

Protein structure prediction models predict the 3D coordinates of a protein backbone given a protein sequence. We measure the network's structure prediction confidence for each protein sequence as a metric for perplexity, which is pLDDT for **Chai-1**, a general protein structure prediction model, and pRMSD for **IgFold**, an antibody-specific structure prediction model. pLDDT measures per-residue confidence, scaled from 0 to 100 with higher scores indicating higher confidence, whereas pRMSD measures the predicted root-mean squared deviation (RMSD) from the hypothetical true crystal structure, where a value approaching 0 indicates less predicted deviation from the crystal.

Physics-based models

bioRxiv preprint doi: https://doi.org/10.64898/2025.12.27.696706; this version posted December 27, 2025. The copyright holder for this preprint (which was not certified by peer review) is the author/funder, who has granted bioRxiv a license to display the preprint in perpetuity. It is made available under aCC-BY 4.0 International license.

We seek to compare the performance of protein AI models versus empirical models of protein energy, which has been a longstanding approach for protein design efforts. **Rosetta**, the classic protein structure prediction and design software, employs an optimized energy function, REF2015, that assesses the energy of atomic interactions within a globular protein. Score functions within Rosetta are composed of weighted sums of various energy terms. Some of these terms correspond to physical forces, such as electrostatics and Van Der Waals interactions, while others represent statistical terms, like the likelihood of observing specific torsion angles in Ramachandran space:

$$E(\{x_i\}_{i=1}^{n}, \text{structure}) = \sum_{t=\{\text{energy types}\}} w_t \, \epsilon_t(\{x_i\}_{i=1}^{n}, \text{structure}) \,, \quad (4)$$

where ϵ_i is a Rosetta energy term, and w_i is the respective weighted number. Rosetta's energy calculation does not directly correspond to physical energy units and are instead expressed in Rosetta energy units (REUs). A lower score indicates a higher likelihood of a structure being closer to the native structure. Structures of all antibody mutants are predicted with Chai-1 prior to calculating Rosetta energy.

We use **Biopython** to compute physicochemical properties of antibody sequences: Aromaticity (the frequency of aromatic residues), Charge at pH 7.4 (the net electric charge of the antibody), Flexibility (backbone flexibility based on empirical parameters from amino acid propensities), Gravy (average hydropathy index), Instability index (an estimate of protein stability), and Isoelectric point (pH at which protein carries no net charge).

Germline model

Antibodies begin as germline-encoded sequences that diversify through somatic mutation as they mature toward recognizing specific pathogens. We seek to compare the likelihoods generated by the model to the edit distance of the antibody sequence from its respective germline. For each antibody sequence, we retrieve the V gene and J gene calls using ANARCI[92]. The V gene encodes frameworks (FRs) 1-3, complementarity determining regions (CDRs) 1-2, and part of CDR3; and the J gene encodes part of CDR3 and FR4. The edit distance between the antibody sequence and the retrieved sequences from the V and J gene calls are calculated via the Levenstein distance.

10.4 Zero-shot results

Table S3: Datasets that pass the 5-significant-model cutoff. Datasets that satisfy the 5-significant-model cutoff and are used for the zero-shot model analyses.

Name	Datapoints	Category
garbinski2023_tm1.csv	86	thermostability
hie2023efficient_MEDIUCA_Tm.csv	7	thermostability
hie2023efficient_REGN10987_Tm.csv	2	thermostability
hie2023efficient_S309_Tm.csv	6	thermostability
hie2023efficient_mAb114_Tm.csv	10	thermostability
hutchinson2023enhancement_top200tm1_igg.csv	28	thermostability
hutchinson2023enhancement_top27tm1_igg.csv	192	thermostability

bioRxiv preprint doi: https://doi.org/10.64898/2025.12.27.696706; this version posted December 27, 2025. The copyright holder for this preprint (which was not certified by peer review) is the author/funder, who has granted bioRxiv a license to display the preprint in perpetuity. It is made available under aCC-BY 4.0 International license.

jain2017biophysical_Tm.csv	137	thermostability
jain2023identifying_Tm.csv	137	thermostability
rosace2023automated_tm1_adalimumab.csv	14	thermostability
rosace2023automated_tm1_golimumab.csv	5	thermostability
shanehsazzadeh2023unlocking_DLS.csv	13	thermostability
sulea2019assisted_sdAb_tm.csv	33	thermostability
tresanco2023nbthermo_tm.csv	672	thermostability
ginkgo2025gdpa1_tm1_nanodsf_avg.csv	237	thermostability
adams2018measuring_exp.csv	10970	expression
garbinski2023_exp.csv	94	expression
jain2017biophysical_HEK.csv	137	expression
koenig2017mutational_er_g6.csv	4275	expression
adams2017measuring_4420-fluorescein_exp_er.csv	10970	expression
jain2017biophyscial_HICRT.csv	137	aggregation
jain2017biophysical_ACSINS.csv	137	aggregation
jain2017biophysical_CSIBLI.csv	137	aggregation
jain2023identifying_ACSINS.csv	137	aggregation
jain2023identifying_CSI.csv	137	aggregation
jain2023identifying_HIC.csv	137	aggregation
jetha2019homology_RT.csv	97	aggregation
shanehsazzadeh2023unlocking_ACSINS.csv	13	aggregation
ginkgo2025gdpa1_sec_pctmonomer_avg.csv	244	aggregation
ginkgo2025gdpa1_smac_rt_avg.csv	244	aggregation
ginkgo2025gdpa1_hic_rt_avg.csv	244	aggregation
ginkgo2025gdpa1_acsins_dLmax_ph74_avg.csv	244	aggregation
ginkgo2025gdpa1_acsins_dLmax_ph60_avg.csv	244	aggregation
kraft2019herapin_relrt.csv	128	aggregation
jain2017biophysical_CICRT.csv	137	aggregation
phillips2021binding_cr9114_h1_kd.csv	32392	binding
phillips2021binding_cr9114_h3_kd.csv	32767	binding
phillips2021binding_cr6261_h1_kd.csv	953	binding
phillips2021binding_cr6261_h9_kd.csv	921	binding
shanker2024unsupervised_Ly1404-BQ.1.1_IC50.csv	50	binding
shanker2024unsupervised_Ly1404-BQ.1.1_Kd.csv	36	binding
shanehsazzadeh2024igdesign_Osocimab-FXI_kd.csv	47	binding
shanehsazzadeh2024igdesign_Tezepelumab-TSLP_kd.csv	127	binding
garbinski2023_kd.csv	81	binding
hie2023efficient_CoV2_S309_Kd.csv	20	binding
hie2023efficient_CoV2omicron_REGN10987_Kd.csv	8	binding
hie2023efficient_MEDIUCA_H1Solomon_Kd.csv	21	binding

bioRxiv preprint doi: https://doi.org/10.64898/2025.12.27.696706; this version posted December 27, 2025. The copyright holder for this preprint (which was not certified by peer review) is the author/funder, who has granted bioRxiv a license to display the preprint in perpetuity. It is made available under aCC-BY 4.0 International license.

hutchinson2023enhancement_singlekd_fab.csv	22	binding
hutchinson2023enhancement_singlekd_igg.csv	23	binding
hutchinson2023enhancement_top200kd_fab.csv	50	binding
hutchinson2023enhancement_top200kd_igg.csv	182	binding
hutchinson2023enhancement_top27kd_fab.csv	28	binding
koenig2017mutational_kd_g6.csv	4275	binding
makowksi2022cooptimization_iso_ant.csv	126	binding
makowski2022cooptimization_igg_ant.csv	96	binding
makowski2022cooptimization_igg_ova.csv	96	binding
makowski2022cooptimization_iso_ova.csv	126	binding
shanehsazzadeh2023_trastuzumab_zero_kd.csv	422	binding
shanehsazzadeh2023unlocking_zerokd_trastuzumab.csv	422	binding
warszawski2019_d44_Kd.csv	2048	binding
zimmerman2020antibody_4420_kd.csv	21	binding
adams2017measuring_4420-fluorescein_kd-titeseq.csv	11052	binding
peterson2024integrated_ab_H1HA_kd.csv	1040	binding
peterson2024integrated_ab_H1HA_binary.csv	1071	binding
erasmus2024aintibody_binding.csv	142	binding
tsuruta2024sarscov2_binary.csv	77003	binding
krawczyk2025naturalantibody_1BJ1_bind.csv	68	binding
krawczyk2025naturalantibody_1MHP_bind.csv	131	binding
krawczyk2025naturalantibody_1N8Z_bind.csv	42	binding
krawczyk2025naturalantibody_1NMB_bind.csv	8	binding
krawczyk2025naturalantibody_1VFB_bind.csv	65	binding
krawczyk2025naturalantibody_1XGR_bind.csv	4	binding
krawczyk2025naturalantibody_1XGT_bind.csv	4	binding
krawczyk2025naturalantibody_1YY9_bind.csv	21	binding
krawczyk2025naturalantibody_3HFM_bind.csv	46	binding
krawczyk2025naturalantibody_5F9W_bind.csv	58	binding
krawczyk2025naturalantibody_5GGS_bind.csv	51	binding
krawczyk2025naturalantibody_5GGV_bind.csv	66	binding
krawczyk2025naturalantibody_6MFP_bind.csv	36	binding
krawczyk2025naturalantibody_6NMS_bind.csv	48	binding
krawczyk2025naturalantibody_6NMU_bind.csv	46	binding
krawczyk2025naturalantibody_7BEJ_bind.csv	34	binding
krawczyk2025naturalantibody_7JMO_bind.csv	60	binding
krawczyk2025naturalantibody_7KF0_bind.csv	36	binding
jain2024assessment_Hen_Lys_kd.csv	31	binding
kothiwal2025htp_DCC_ec50.csv	23	binding
kothiwal2025htp_DKK¬†1.00_ec50.csv	18	binding

bioRxiv preprint doi: https://doi.org/10.64898/2025.12.27.696706; this version posted December 27, 2025. The copyright holder for this preprint (which was not certified by peer review) is the author/funder, who has granted bioRxiv a license to display the preprint in perpetuity. It is made available under aCC-BY 4.0 International license.

kothiwal2025htp_IL23R_ec50.csv	56	binding
kothiwal2025htp_PDL1_spr.csv	29	binding
kothiwal2025htp_PDL2_spr.csv	23	binding
kothiwal2025htp_ROBO1_ec50.csv	45	binding
kothiwal2025htp_ROBO1_spr.csv	39	binding
kothiwal2025htp_ROBO2N_hROBO2N_spr.csv	22	binding
kothiwal2025htp_TIGIT_spr.csv	22	binding
jain2023identifying_FcRnRelRT3.csv	132	pharmacokinetics
jain2023identifying_HEPRT3.csv	130	pharmacokinetics
makowski2022cooptimization_pI.csv	126	pharmacokinetics
jain2017biophysical_BVPELISA.csv	137	polyreactivity
jain2017biophysical_SMACRT.csv	137	polyreactivity
jain2023identifying_BVP.csv	137	polyreactivity
jain2023identifying_CIC.csv	137	polyreactivity
jain2023identifying_FVC32.csv	115	polyreactivity
jain2023identifying_FvFVIII2.csv	115	polyreactivity
jain2023identifying_FvLysM2.csv	115	polyreactivity
jain2023identifying_SMAC.csv	137	polyreactivity
shanker2024unsupervised_APC-MFI_Elotuzumab-Ixekuzimab.csv	6	polyreactivity
shanker2024unsupervised_APC-MFI_Ly1404.csv	18	polyreactivity
ginkgo2025gdpa1_hac_rt_avg.csv	94	polyreactivity
ginkgo2025gdpa1_polyreactivity_prscore_cho_avg.csv	197	polyreactivity
marks2021humanization_immunogenicity.csv	217	immunogenicity
naturalantibody2025therapeutics_ada_prevalence_all.csv	3456	immunogenicity
naturalantibody2025therapeutics_ada_incidence_all.csv	820	immunogenicity
naturalantibody2025therapeutics_ada_baseline_all.csv	579	immunogenicity

Table S4: **Zero-shot performance summary across all models**. Tabulated correlation between model confidence and actual fitness score, averaged across all models. Some of the assay labels are inverted so that a positive correlation between fitness and assay value always indicates improved model performance.

All Categories	Tm.	Exp.	Agg.	Bind.	PK.	Poly.	Imm.
0.01	0.06	**0.22**	-0.07	0.05	-0.06	0.02	0.07

Table S5: Zero-shot performance summary for each individual model. Tabulated correlation between model confidence and actual fitness score, for each model.

Scoring Method	All Categories	Tm.	Exp.	Agg.	Bind.	PK.	Poly.	Imm.
IgLM	-0.01	-0.05	0.17	-0.16	0.08	-0.18	-0.02	0.21
ProGen2 Small	0.00	0.13	0.39	-0.15	0.17	-0.05	-0.07	0.10
ProGen2 Medium	0.08	0.09	0.36	-0.19	**0.18**	-0.13	-0.03	**0.34**
ProGen2 Base	-0.02	0.02	0.38	-0.15	0.01	-0.16	-0.02	0.16

bioRxiv preprint doi: https://doi.org/10.64898/2025.12.27.696706; this version posted December 27, 2025. The copyright holder for this preprint (which was not certified by peer review) is the author/funder, who has granted bioRxiv a license to display the preprint in perpetuity. It is made available under aCC-BY 4.0 International license.

ProGen2 OAS	0.04	0.09	0.12	-0.18	0.11	-0.18	-0.03	0.12
ProGen2 BFD90	-0.02	0.14	0.35	-0.17	0.12	-0.15	-0.04	0.33
ProGen2 Large	0.00	0.01	0.36	-0.15	0.09	-0.13	-0.04	0.17
ProGen2 XLarge	-0.01	0.07	0.32	-0.15	0.10	-0.19	-0.03	0.28
AntiBERTy	0.00	-0.14	0.24	-0.11	0.06	-0.16	0.00	0.17
ESM2 8M	-0.04	0.01	0.26	-0.08	-0.03	-0.10	-0.05	-0.10
ESM2 35M	-0.04	0.14	0.30	-0.10	-0.06	-0.10	-0.02	-0.14
ESM2 150M	-0.04	0.00	**0.44**	-0.12	0.01	-0.15	-0.07	-0.03
ESM2 650M	0.04	0.15	0.37	-0.07	0.12	-0.07	0.04	-0.01
ESM2 3B	0.03	0.16	0.34	-0.05	0.14	-0.12	0.02	-0.04
ISM 650M	0.06	0.16	0.34	-0.11	0.13	-0.12	0.07	0.01
ISM 3B	0.06	0.05	0.38	-0.08	0.13	-0.12	0.08	0.09
ESM IF	0.01	0.25	0.29	-0.05	-0.04	-0.08	0.08	0.10
ProteinMPNN	0.04	-0.05	0.09	-0.07	0.04	-0.08	0.09	0.08
AbMPNN	0.00	-0.08	0.14	-0.11	0.06	-0.12	0.13	0.07
Chai 1	0.01	**0.45**	0.38	-0.17	0.01	-0.12	0.04	0.11
IgFold	**0.12**	0.08	0.23	-0.17	0.18	-0.16	0.16	0.08
PyRosetta	-0.02	-0.01	-0.13	-0.04	0.07	-0.06	-0.04	0.03
BP aromaticity	0.06	0.01	0.07	0.10	0.04	0.11	0.15	-0.02
BP charge at pH 7.4	0.00	-0.08	0.05	**0.29**	-0.11	**0.59**	**0.18**	0.01
BP flexibility	-0.07	0.11	-0.14	-0.13	-0.03	-0.23	0.00	0.02
BP gravy	0.04	0.02	0.05	-0.01	0.06	-0.08	-0.05	0.03
BP instability index	-0.06	0.04	0.07	0.13	-0.15	0.15	0.00	-0.14
BP isoelectric point	0.01	-0.08	0.04	0.18	-0.13	0.54	0.16	0.04
BP molecular weight	0.02	-0.04	0.11	0.08	-0.06	0.06	0.06	-0.08
LD from GL	0.00	0.04	0.14	-0.18	0.11	-0.19	-0.07	0.19

Table S6: Zero-shot performance across architectures. Autoregressive language models include IgLM and the ProGen2 suite. Masked language models include AntiBERTy, the ESM2 suite, and the ISM suite. Inverse folding models include AbMPNN, ESM IF, and ProteinMPNN. Structure predictors include Chai-1 and IgFold. Sequence-based physics models include BioPython. Structure-based physics models include PyRosetta.

Architecture	All Categories	Tm.	Exp.	Agg.	Bind.	PK.	Poly.	Imm.
Autoregressive LMs	0.01	0.06	0.31	-0.16	**0.11**	-0.15	-0.03	**0.21**
Masked LMs	0.01	0.07	**0.33**	-0.09	0.06	-0.12	0.01	-0.01
Inverse folding	0.02	0.04	0.17	-0.08	0.02	-0.09	**0.10**	0.08
Structure predictors	**0.07**	**0.27**	0.30	-0.17	0.09	-0.14	**0.10**	0.10
Seq-based physics	0.00	0.00	0.04	**0.09**	-0.05	**0.16**	0.07	-0.02
Struc-based physics	-0.02	-0.01	-0.13	-0.04	0.07	-0.06	-0.04	0.03

Table S7: Zero-shot performance across sequence-only and structure-informed models. Masked language modes without structure are AntiBERTY and the ESM2 suite. Masked language models with structure include the ISM suite. Inverse folding models are ESM-IF, ProteinMPNN, and AbMPNN.

bioRxiv preprint doi: https://doi.org/10.64898/2025.12.27.696706; this version posted December 27, 2025. The copyright holder for this preprint (which was not certified by peer review) is the author/funder, who has granted bioRxiv a license to display the preprint in perpetuity. It is made available under aCC-BY 4.0 International license.

Structure predictors are Chai-1 and IgFold. Sequence-only masked language and causal language models are the ESM2 suite, AntiBERTY, IgLM, and ProGen2.

Learned rep.	All Categories	Tm.	Exp.	Agg.	Bind.	PK.	Poly.	Imm.
MLMs w/o structure	-0.01	0.05	0.33	-0.09	0.04	-0.12	-0.01	-0.02
MLM w/ structure	0.06	0.11	**0.36**	-0.10	**0.13**	-0.12	0.08	0.05
Inverse folding	0.02	0.04	0.17	**-0.08**	0.02	**-0.09**	**0.10**	0.08
Structure predictor	**0.07**	**0.27**	0.30	-0.17	0.09	-0.14	**0.10**	**0.10**
MLMs and CLMs	0.01	0.06	0.32	-0.13	0.09	-0.13	-0.01	**0.10**

Table S8: Zero-shot performance across general protein and antibody-specific AI models. General protein AI models include the ProGen2 suite, the ESM2 suite, the ISM suite, ESM IF, ProteinMPNN, and Chai-1. Antibody specific models include IgLM, AntiBERTy, AbMPNN, and IgFold.

Protein family	All categories	Tm.	Exp.	Agg.	Bind.	PK.	Poly.	Imm.
General protein	0.01	**0.11**	**0.32**	**-0.12**	0.07	**-0.12**	0.00	0.09
Antibody specific	**0.03**	-0.05	0.19	-0.14	**0.09**	-0.16	**0.07**	**0.13**

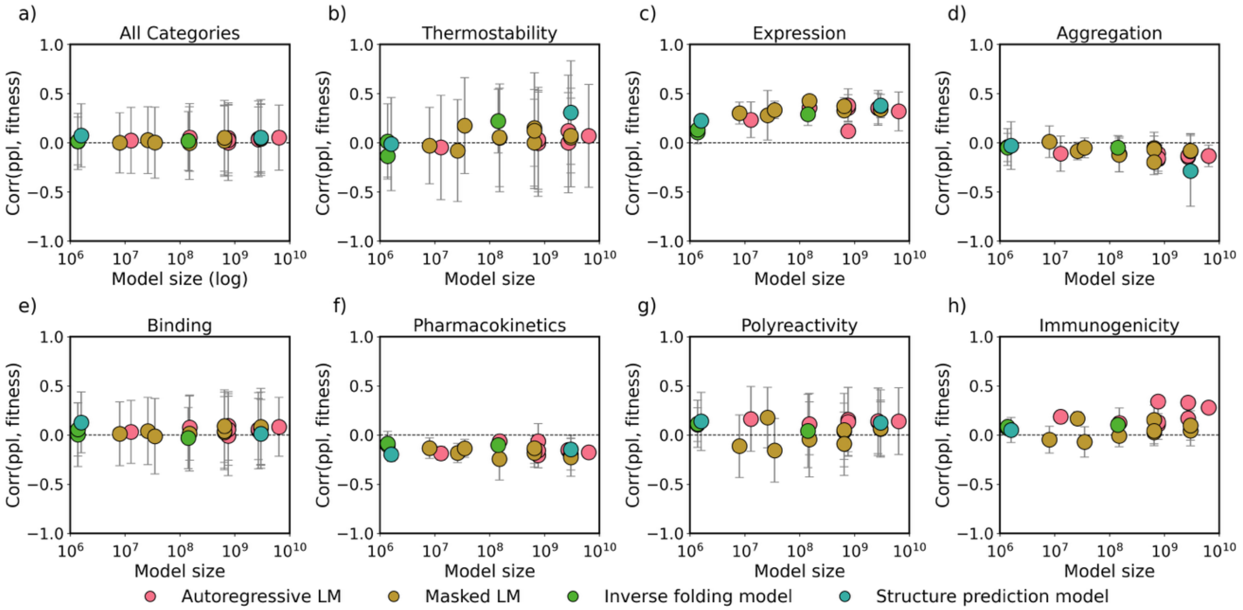

Figure S2: Distribution of zero-shot prediction performances for each model compared to parameter size. Models are colored based on their architecture, and the y-axis displays the range of Spearman's correlations between model confidence and developability label, where a correlation approaching 1.0 is ideal. We only report Spearman's correlations with datasets that exhibit at least five significant dataset-model p values to ensure our conclusions are statistically sound.

bioRxiv preprint doi: https://doi.org/10.64898/2025.12.27.696706; this version posted December 27, 2025. The copyright holder for this preprint (which was not certified by peer review) is the author/funder, who has granted bioRxiv a license to display the preprint in perpetuity. It is made available under aCC-BY 4.0 International license.

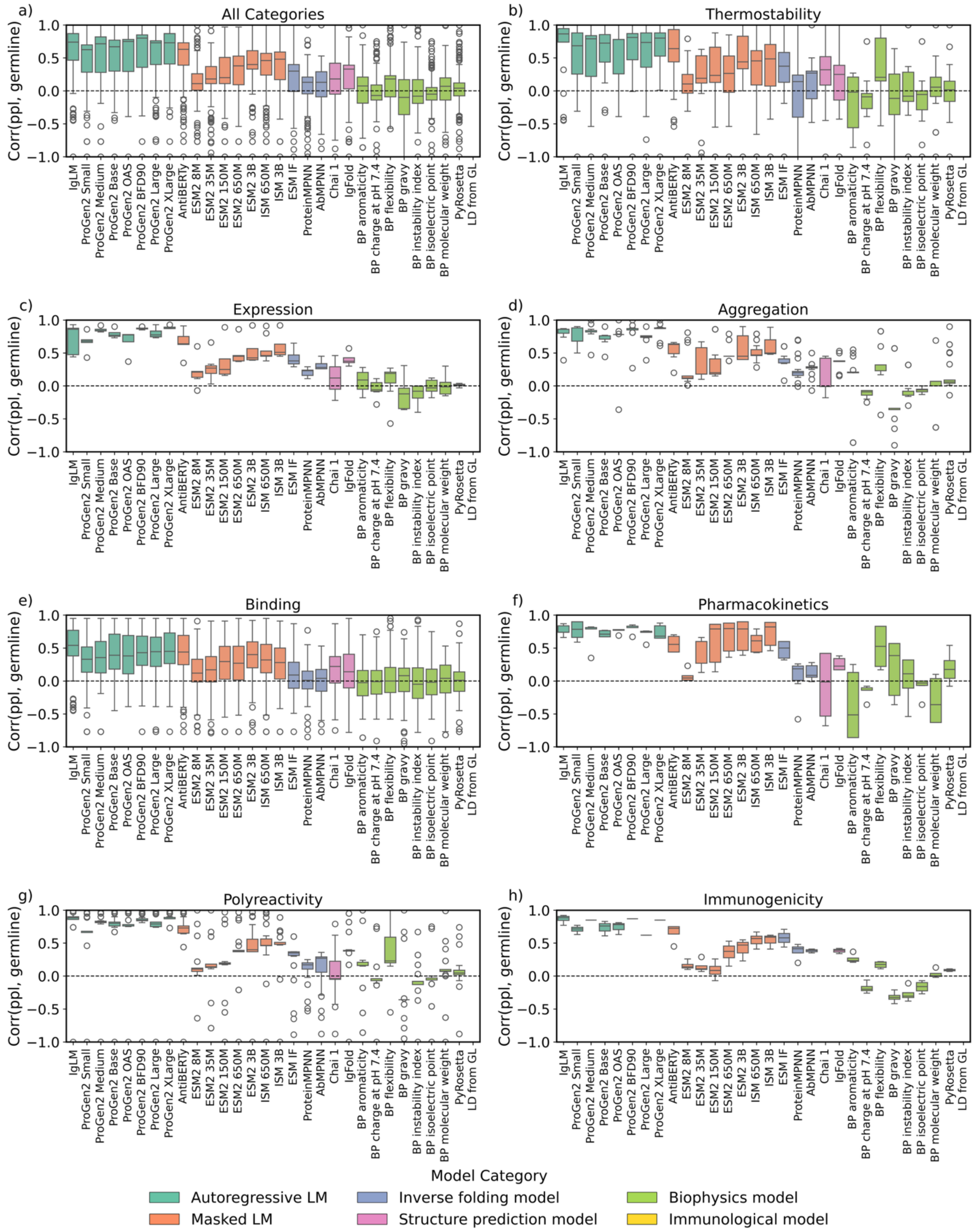

Figure S3: Distribution of zero-shot prediction correlations with germline. Models are colored based on their architecture, and the *y*-axis displays the range of Spearman's correlations between model confidence and distance from germline. A correlation approaching 1.0 indicates the model is highly

bioRxiv preprint doi: https://doi.org/10.64898/2025.12.27.696706; this version posted December 27, 2025. The copyright holder for this preprint (which was not certified by peer review) is the author/funder, who has granted bioRxiv a license to display the preprint in perpetuity. It is made available under aCC-BY 4.0 International license.

confident in germline sequences and highly unconfident in sequences far from germline. We only report Spearman's correlations with datasets that exhibit at least five significant dataset-model p-values to ensure our conclusions are statistically sound.

Table S9: Zero-shot correlation with germline signal. Tabulated correlation between model confidence and distance from germline. A correlation approaching 1.0 indicates the model is highly confident in germline sequences and highly unconfident in sequences far from germline.

Scoring Method	All Categories	Tm.	Exp.	Agg.	Bind.	PK.	Poly.	Imm.
IgLM	0.74	**0.87**	0.86	0.86	**0.54**	0.74	**0.89**	**0.89**
ProGen2 Small	0.63	0.69	0.68	0.68	0.33	0.79	0.68	0.72
ProGen2 Medium	0.72	0.79	0.84	0.82	0.36	0.79	0.82	0.85
ProGen2 Base	0.67	0.73	0.77	0.76	0.39	0.71	0.80	0.76
ProGen2 OAS	0.75	0.78	0.78	0.78	0.38	0.78	0.78	0.78
ProGen2 BFD90	**0.80**	0.81	0.87	0.85	0.43	0.81	0.85	0.87
ProGen2 Large	0.73	0.74	0.78	0.75	0.45	0.74	0.79	0.62
ProGen2 XLarge	0.73	0.80	**0.88**	**0.88**	0.45	0.68	0.87	0.85
AntiBERTy	0.63	0.64	0.64	0.63	0.44	0.56	0.73	0.73
ESM2 8M	0.11	0.11	0.21	0.12	0.12	0.05	0.11	0.14
ESM2 35M	0.18	0.19	0.27	0.18	0.17	0.60	0.14	0.11
ESM2 150M	0.20	0.24	0.25	0.20	0.30	0.79	0.19	0.08
ESM2 650M	0.38	0.27	0.45	0.45	0.27	0.79	0.38	0.38
ESM2 3B	0.40	0.44	0.43	0.45	0.40	0.79	0.40	0.47
ISM 650M	0.46	0.46	0.46	0.47	0.32	0.61	0.46	0.57
ISM 3B	0.48	0.49	0.51	0.50	0.28	**0.82**	0.48	0.59
ESM IF	0.30	0.38	0.38	0.35	0.09	0.50	0.35	0.58
ProteinMPNN	0.13	0.14	0.23	0.20	0.01	0.19	0.17	0.42
AbMPNN	0.13	0.27	0.28	0.28	0.04	0.08	0.27	0.39
Chai 1	0.18	0.32	0.12	0.42	0.22	-0.02	-0.04	0.43
IgFold	0.33	0.25	0.38	0.38	0.14	0.23	0.38	0.39
PyRosetta	0.04	0.02	0.02	0.06	0.01	0.18	0.05	0.09
BP aromaticity	0.08	-0.02	0.09	0.21	-0.03	-0.52	0.16	0.24
BP charge at pH 7.4	-0.07	-0.09	-0.06	-0.09	-0.01	-0.14	-0.07	-0.20
BP flexibility	0.18	0.21	0.19	0.23	0.00	0.53	0.23	0.18
BP gravy	-0.10	-0.11	-0.12	-0.36	0.08	0.39	-0.36	-0.33
BP instability index	-0.08	-0.08	-0.08	-0.08	-0.05	0.11	-0.08	-0.31
BP isoelectric point	-0.05	-0.06	-0.03	-0.06	-0.02	-0.03	-0.06	-0.16
BP molecular weight	0.07	0.06	-0.02	0.07	0.04	-0.36	0.08	0.01
LD from GL	1.00	1.00	1.00	1.00	1.00	1.00	1.00	1.00

Table S10: Zero-shot correlation to germline signal across sequence-only and structure-informed models. Masked language modes without structure are AntiBERTY and the ESM2 suite. Masked language models with structure include the ISM suite. Inverse folding models are ESM-IF, ProteinMPNN, and AbMPNN. Structure predictors are Chai-1 and IgFold. Sequence-only masked language and causal language models are the ESM2 suite, AntiBERTY, IgLM, and ProGen2.

bioRxiv preprint doi: https://doi.org/10.64898/2025.12.27.696706; this version posted December 27, 2025. The copyright holder for this preprint (which was not certified by peer review) is the author/funder, who has granted bioRxiv a license to display the preprint in perpetuity. It is made available under aCC-BY 4.0 International license.

Learned rep.	All Categories	Tm.	Exp.	Agg.	Bind.	PK.	Poly.	Imm.
MLMs w/o structure	0.32	0.31	0.37	0.34	0.28	0.60	0.32	0.32
MLM w/ structure	0.47	0.47	0.49	0.49	0.30	**0.72**	0.47	0.58
Inverse folding	0.19	0.26	0.30	0.28	0.05	0.26	0.26	0.46
Structure predictor	0.26	0.29	0.25	0.40	0.18	0.11	0.17	0.41
MLMs and CLMs	**0.54**	**0.56**	**0.60**	**0.59**	**0.35**	0.69	**0.59**	**0.59**

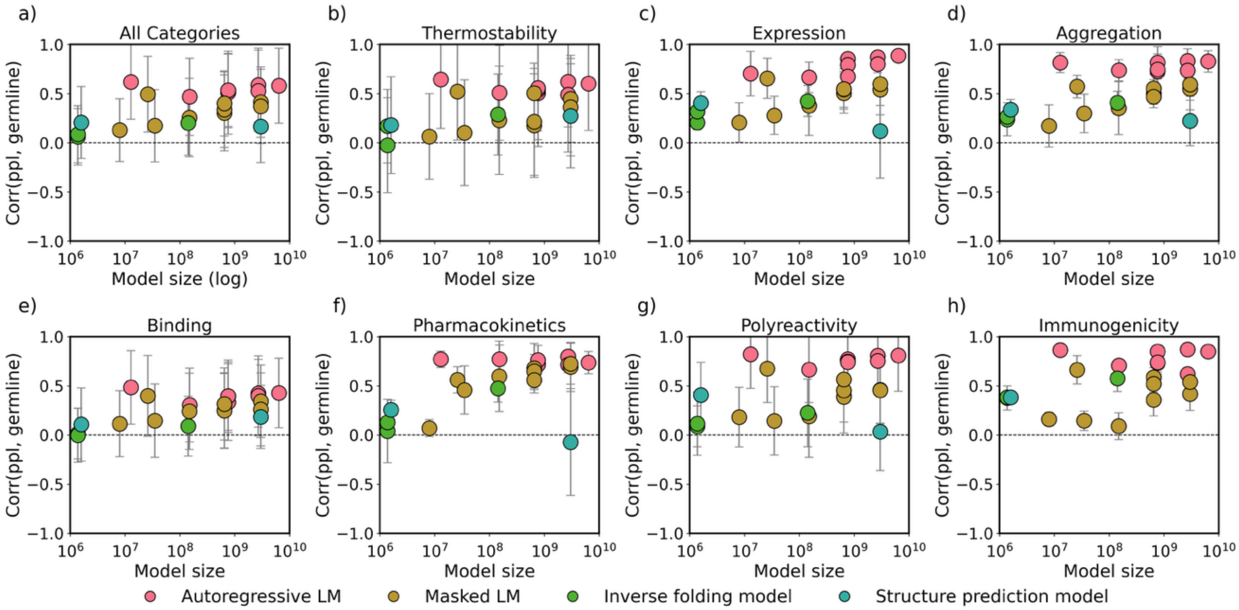

Figure S4: Zero-shot prediction correlations with germline compared to parameter size. Models are colored based on their architecture, and the *y*-axis displays the range of Spearman's correlations between model confidence and the distance from germline. A correlation approaching 1.0 indicates the model is highly confident in germline sequences and highly unconfident in sequences far from germline. We only report Spearman's correlations with datasets that exhibit at least five significant dataset-model p-values to ensure our conclusions are statistically sound.

bioRxiv preprint doi: https://doi.org/10.64898/2025.12.27.696706; this version posted December 27, 2025. The copyright holder for this preprint (which was not certified by peer review) is the author/funder, who has granted bioRxiv a license to display the preprint in perpetuity. It is made available under aCC-BY 4.0 International license.

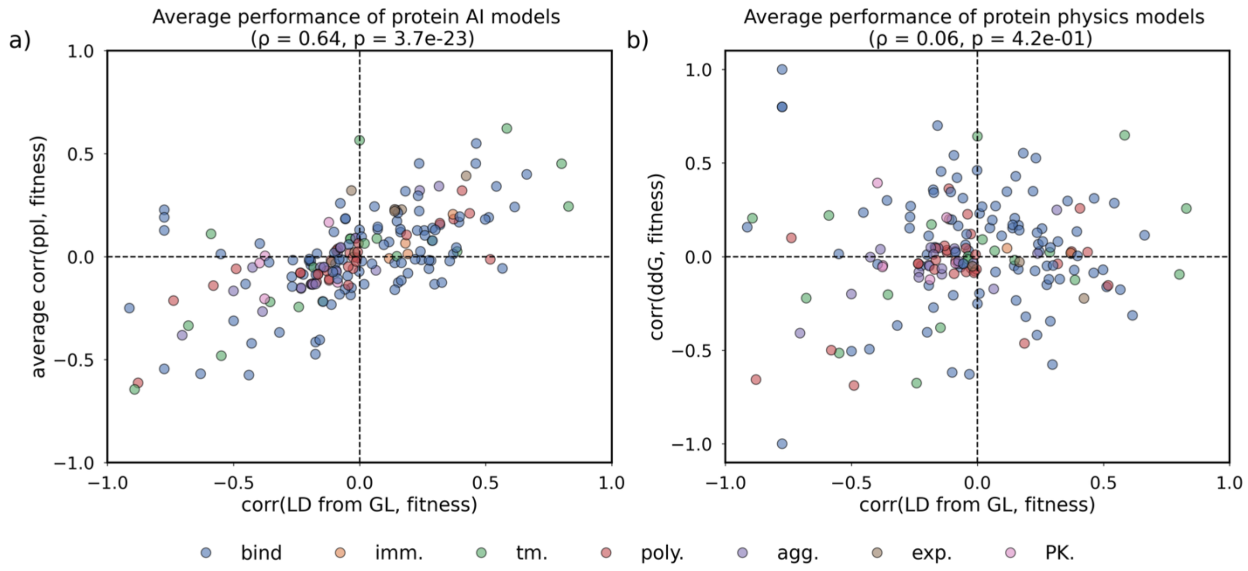

Figure S5: Average zero-shot prediction performance across datasets with varying directions of germline and fitness. Each point represents one developability dataset. The *x*-axis is the correlation between the distance from germline and the fitness, where a correlation approaching -1.0 indicates the sequences closer to germline have lower fitness, and a correlation approaching 1.0 indicates the sequences closer to germline have higher fitness. The *y*-axis is the average correlation of perplexity to fitness across all benchmarked protein AI models. a) Protein AI models, and b) PyRosetta. We only report Spearman's correlations with five significant dataset-model p-values to ensure our conclusions are statistically sound.

Partial correlation calculation

The partial correlation coefficient[60] measures the relationship between two variables (A, B) while eliminating influence of a third variable C.

$$r_{AB,C} = \frac{r_{AB} - r_{AC}\, r_{BC}}{\sqrt{1 - r_{AC}^2}\sqrt{1 - r_{BC}^2}}$$

We calculate the partial correlation between the protein AI model perplexity (A) and the developability label (B) accounting for germline signal (C). For a given developability dataset, we calculate the Spearman's correlations between the AI model perplexity and the developability label (r_{AB}), between the AI model and germline signal (r_{AC}), and between the developability label and germline signal (r_{BC}).

Table S11: Partial correlation datasets. Datasets for which at least five models show statistically significant correlations and for which the mean Spearman correlation across models is at least 0.3.

Name	Datapoints	Category
hie2023efficient_MEDIUCA_Tm.csv	7	thermostability
hie2023efficient_REGN10987_Tm.csv	2	thermostability
hie2023efficient_S309_Tm.csv	6	thermostability
hutchinson2023enhancement_multitm1_igg.csv	15	thermostability
rosace2023automated_tm1_adalimumab.csv	14	thermostability
rosace2023automated_tm1_golimumab.csv	5	thermostability

bioRxiv preprint doi: https://doi.org/10.64898/2025.12.27.696706; this version posted December 27, 2025. The copyright holder for this preprint (which was not certified by peer review) is the author/funder, who has granted bioRxiv a license to display the preprint in perpetuity. It is made available under aCC-BY 4.0 International license.

shanehsazzadeh2023unlocking_DLS.csv	13	thermostability
shanehsazzadeh2023unlocking_DSF1.csv	13	thermostability
garbinski2023_exp.csv	94	expression
shanehsazzadeh2023unlocking_ACSINS.csv	13	aggregation
shanehsazzadeh2023unlocking_NRCGE.csv	13	aggregation
shanehsazzadeh2023unlocking_SEC.csv	13	aggregation
garbinski2023_kd.csv	81	binding
hie2023efficient_CoV2_S309_Kd.csv	20	binding
hie2023efficient_CoV2omicron_REGN10987_Kd.csv	8	binding
hie2023efficient_MEDIUCA_H1Solomon_Kd.csv	21	binding
hie2023efficient_MEDIUCA_H4Hubei_Kd.csv	12	binding
hutchinson2023enhancement_multikd_fab.csv	15	binding
kothiwal2025htp_DCC_ec50.csv	23	binding
kothiwal2025htp_PDL2_spr.csv	23	binding
kothiwal2025htp_ROBO2N_hROBO2N_spr.csv	22	binding
kothiwal2025htp_TIGIT_spr.csv	22	binding
krawczyk2025naturalantibody_1DQJ_bind.csv	20	binding
krawczyk2025naturalantibody_1NMB_bind.csv	8	binding
krawczyk2025naturalantibody_2NYY_bind.csv	20	binding
krawczyk2025naturalantibody_3N85_bind.csv	9	binding
krawczyk2025naturalantibody_5GGV_bind.csv	66	binding
krawczyk2025naturalantibody_7JMO_bind.csv	60	binding
krawczyk2025naturalantibody_7KF0_bind.csv	36	binding
makowksi2022cooptimization_iso_ant.csv	126	binding
shanehsazzadeh2023unlocking_adcc_ec50.csv	13	binding
shanehsazzadeh2023unlocking_kd_hher2_mab.csv	13	binding
shanker2024unsupervised_Ly1404-BQ.1.1_IC50.csv	50	binding
shanker2024unsupervised_Ly1404-BQ.1.1_Kd.csv	36	binding
shanker2024unsupervised_SA58-BA.1_IC50.csv	19	binding
shanker2024unsupervised_SA58-BQ.1.1_Kd.csv	7	binding
jain2023identifying_FVC32.csv	115	polyreactivity
jain2023identifying_FvFVIII2.csv	115	polyreactivity
jain2023identifying_FvLysM2.csv	115	polyreactivity
rosace2023automated_CICRT1_golimumab.csv	5	polyreactivity
rosace2023automated_CICRT2_golimumab.csv	5	polyreactivity
shanker2024unsupervised_APC-MFI_Ly1404.csv	18	polyreactivity
shanker2024unsupervised_APC-MFI_SA58.csv	15	polyreactivity

Table S12: Partial correlations of protein AI models after accounting for germline signal.

Scoring Method	Original corr.	Adjusted corr.	Diff.

bioRxiv preprint doi: https://doi.org/10.64898/2025.12.27.696706; this version posted December 27, 2025. The copyright holder for this preprint (which was not certified by peer review) is the author/funder, who has granted bioRxiv a license to display the preprint in perpetuity. It is made available under aCC-BY 4.0 International license.

IgLM	0.50	0.26	0.47
ProGen2 Small	0.47	0.29	0.38
ProGen2 Medium	0.46	0.23	0.51
ProGen2 Base	0.48	0.30	0.37
ProGen2 OAS	0.48	0.23	0.52
ProGen2 BFD90	0.48	0.23	0.52
ProGen2 Large	0.49	0.21	0.57
ProGen2 XLarge	0.47	0.17	**0.63**
AntiBERTy	0.49	0.30	0.39
ESM2 8M	0.46	0.39	0.16
ESM2 35M	0.53	0.38	0.27
ESM2 150M	**0.54**	0.43	0.21
ESM2 650M	0.50	0.35	0.31
ESM2 3B	0.49	0.32	0.35
ISM 650M	0.50	0.35	0.30
ISM 3B	0.52	0.37	0.28
ESM IF	0.46	0.38	0.18
ProteinMPNN	0.51	0.46	0.09
AbMPNN	0.51	0.41	0.20
Chai 1	0.53	0.42	0.20
IgFold	0.52	0.36	0.31
PyRosetta	0.48	0.40	0.16
BP aromaticity	0.48	0.40	0.16
BP charge at pH 7.4	0.48	0.45	0.06
BP flexibility	0.49	0.37	0.25
BP instability index	0.49	0.45	0.09
BP isoelectric point	0.51	**0.48**	0.05
BP molecular weight	0.46	0.38	0.17

10.5 Few-shot results

Table S13: Datasets that pass the 5-significant-model cutoff. Datasets that satisfy the 5-significant-model cutoff and are used for the few-shot model analyses.

Name	Datapoints	Category
tresanco2023nbthermo_tm.csv	672	thermostability
adams2018measuring_exp.csv	10970	expression
koenig2017mutational_er_g6.csv	4275	expression
adams2017measuring_4420-fluorescein_exp_er.csv	10970	expression
ginkgo2025gdpa1_acsins_dLmax_ph74_avg.csv	244	aggregation
phillips2021binding_cr9114_h1_kd.csv	32392	binding
phillips2021binding_cr9114_h3_kd.csv	32767	binding
phillips2021binding_cr6261_h1_kd.csv	953	binding
phillips2021binding_cr6261_h9_kd.csv	921	binding
engelhart2022dataset_scFv-SARS-CoV-2_affinity.csv	352140	binding

bioRxiv preprint doi: https://doi.org/10.64898/2025.12.27.696706; this version posted December 27, 2025. The copyright holder for this preprint (which was not certified by peer review) is the author/funder, who has granted bioRxiv a license to display the preprint in perpetuity. It is made available under aCC-BY 4.0 International license.

shanehsazzadeh2024igdesign_Tezepelumab-TSLP_kd.csv	127	binding
koenig2017mutational_kd_g6.csv	4275	binding
makowksi2022cooptimization_iso_ant.csv	126	binding
shanehsazzadeh2023_trastuzumab_zero_kd.csv	422	binding
shanehsazzadeh2023unlocking_zerokd_trastuzumab.csv	422	binding
warszawski2019_d44_Kd.csv	2048	binding
adams2017measuring_4420-fluorescein_kd-titeseq.csv	11052	binding
peterson2024integrated_ab_H1HA_kd.csv	1040	binding
peterson2024integrated_ab_H1HA_binary.csv	1071	binding
tsuruta2024avida-hIL6_binary.csv	573891	binding
tsuruta2024sarscov2_binary.csv	77003	binding
naturalantibody2025therapeutics_ada_prevalence_all.csv	3456	immunogenicity
naturalantibody2025therapeutics_ada_incidence_all.csv	820	immunogenicity
naturalantibody2025therapeutics_ada_baseline_all.csv	579	immunogenicity

bioRxiv preprint doi: https://doi.org/10.64898/2025.12.27.696706; this version posted December 27, 2025. The copyright holder for this preprint (which was not certified by peer review) is the author/funder, who has granted bioRxiv a license to display the preprint in perpetuity. It is made available under aCC-BY 4.0 International license.

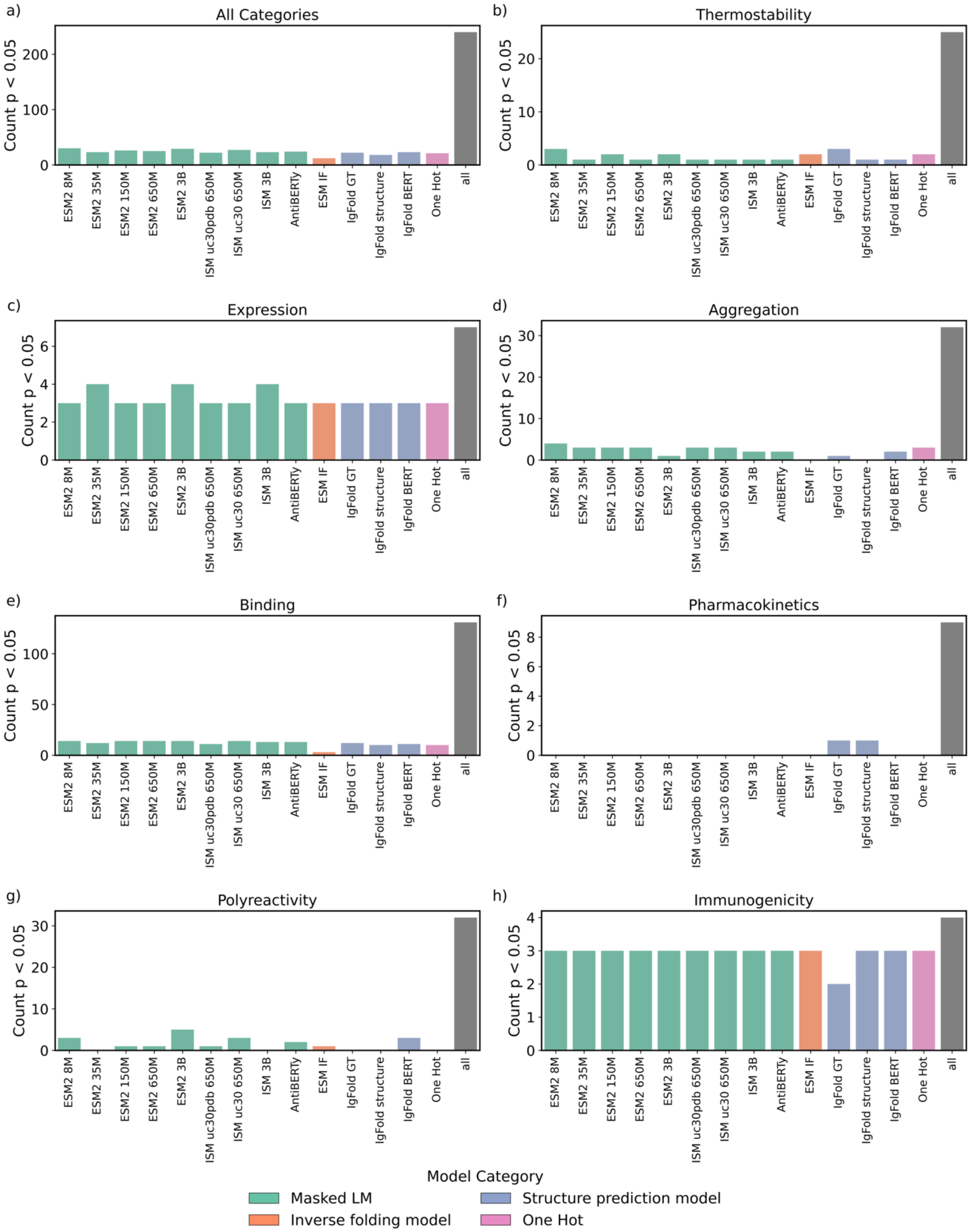

Figure S6: Count of significant correlations per few-shot model. Count of significant (p-value < 0.05) correlations each protein AI model had within each developability category. We only report Spearman's correlations with five significant dataset-model p values to ensure our conclusions are statistically sound.

bioRxiv preprint doi: https://doi.org/10.64898/2025.12.27.696706; this version posted December 27, 2025. The copyright holder for this preprint (which was not certified by peer review) is the author/funder, who has granted bioRxiv a license to display the preprint in perpetuity. It is made available under aCC-BY 4.0 International license.

Table S14: **Few-shot performance summary across all models**. Tabulated correlation between model confidence and actual fitness score, averaged across all models. Some of the assay labels are inverted so that a positive correlation between fitness and assay value always indicates improved model performance.

All Categories	Tm.	Exp.	Agg.	Bind.	Imm.
0.49	**0.61**	0.53	0.32	0.43	0.44

Table S15: Few-shot performance summary for each individual model. Tabulated correlation between model confidence and actual fitness score, for each model.

Scoring Method	All Categories	Tm.	Exp.	Agg.	Bind.	Imm.
AntiBERTy	0.54	0.60	0.55	0.52	0.47	0.45
ESM2 8M	0.51	0.57	0.55	0.25	0.39	0.47
ESM2 35M	0.50	0.70	**0.56**	0.28	0.42	0.47
ESM2 150M	0.55	0.61	0.55	0.48	0.52	**0.49**
ESM2 650M	0.51	0.63	0.54	0.40	0.48	0.43
ESM2 3B	**0.56**	0.63	0.54	0.27	0.56	0.44
ISM uc30 650M	0.55	**0.75**	0.55	0.42	0.55	0.44
ISM uc30pdb 650M	0.54	0.72	0.55	**0.66**	0.45	0.45
ISM 3B	**0.56**	0.74	**0.56**	0.37	**0.61**	0.45
ESM IF	0.29	0.50	0.46	0.18	0.20	0.47
IgFold BERT	0.48	0.54	0.55	0.43	0.41	0.47
IgFold GT	0.40	0.51	0.47	-0.03	0.33	0.40
IgFold structure	0.41	0.51	0.54	-0.03	0.37	0.34
One Hot	0.42	0.51	0.47	0.32	0.30	0.36

Table S16: Few-shot performance across architectures. Masked language models include AntiBERTy, the ESM2 suite, and the ISM suite. Inverse folding models include ESM IF. Structure predictors include IgFold.

Architecture	All Categories	Tm.	Exp.	Agg.	Bind.	Imm.
Masked LMs	**0.54**	**0.66**	**0.55**	**0.41**	**0.49**	0.45
Inverse folding	0.29	0.50	0.46	0.18	0.20	**0.47**
Structure predictors	0.43	0.52	0.52	0.12	0.37	0.40
One Hot	0.42	0.51	0.47	0.32	0.30	0.36

Table S17: Few-shot performance across sequence-only and structure-informed models. Masked language modes without structure are AntiBERTY and the ESM2 suite. Masked language models with structure include the ISM suite. Inverse folding models are ESM-IF, ProteinMPNN, and AbMPNN. Structure predictors are Chai-1 and IgFold. Sequence-only masked language and causal language models are the ESM2 suite, AntiBERTY, IgLM, and ProGen2.

Architecture	All Categories	Tm.	Exp.	Agg.	Bind.	Imm.
MLM w/o structure	0.53	0.62	0.55	0.37	0.47	0.46
MLM w/ structure	**0.55**	**0.74**	**0.55**	**0.48**	**0.54**	0.45

bioRxiv preprint doi: https://doi.org/10.64898/2025.12.27.696706; this version posted December 27, 2025. The copyright holder for this preprint (which was not certified by peer review) is the author/funder, who has granted bioRxiv a license to display the preprint in perpetuity. It is made available under aCC-BY 4.0 International license.

Inverse folding	0.29	0.50	0.46	0.18	0.20	**0.47**
Structure predictors	0.43	0.52	0.52	0.12	0.37	0.40
One Hot	0.42	0.51	0.47	0.32	0.30	0.36

Table S18: Few-shot performance across general protein and antibody-specific AI models. General protein AI models include the ESM2 suite, the ISM suite, and ESM IF. Antibody specific models include AntiBERTy and IgFold.

Protein family	All Categories	Tm.	Exp.	Agg.	Bind.	Imm.
General protein	**0.51**	**0.65**	**0.54**	**0.37**	**0.46**	**0.46**
Antibody specific	0.46	0.54	0.53	0.22	0.39	0.42

bioRxiv preprint doi: https://doi.org/10.64898/2025.12.27.696706; this version posted December 27, 2025. The copyright holder for this preprint (which was not certified by peer review) is the author/funder, who has granted bioRxiv a license to display the preprint in perpetuity. It is made available under aCC-BY 4.0 International license.

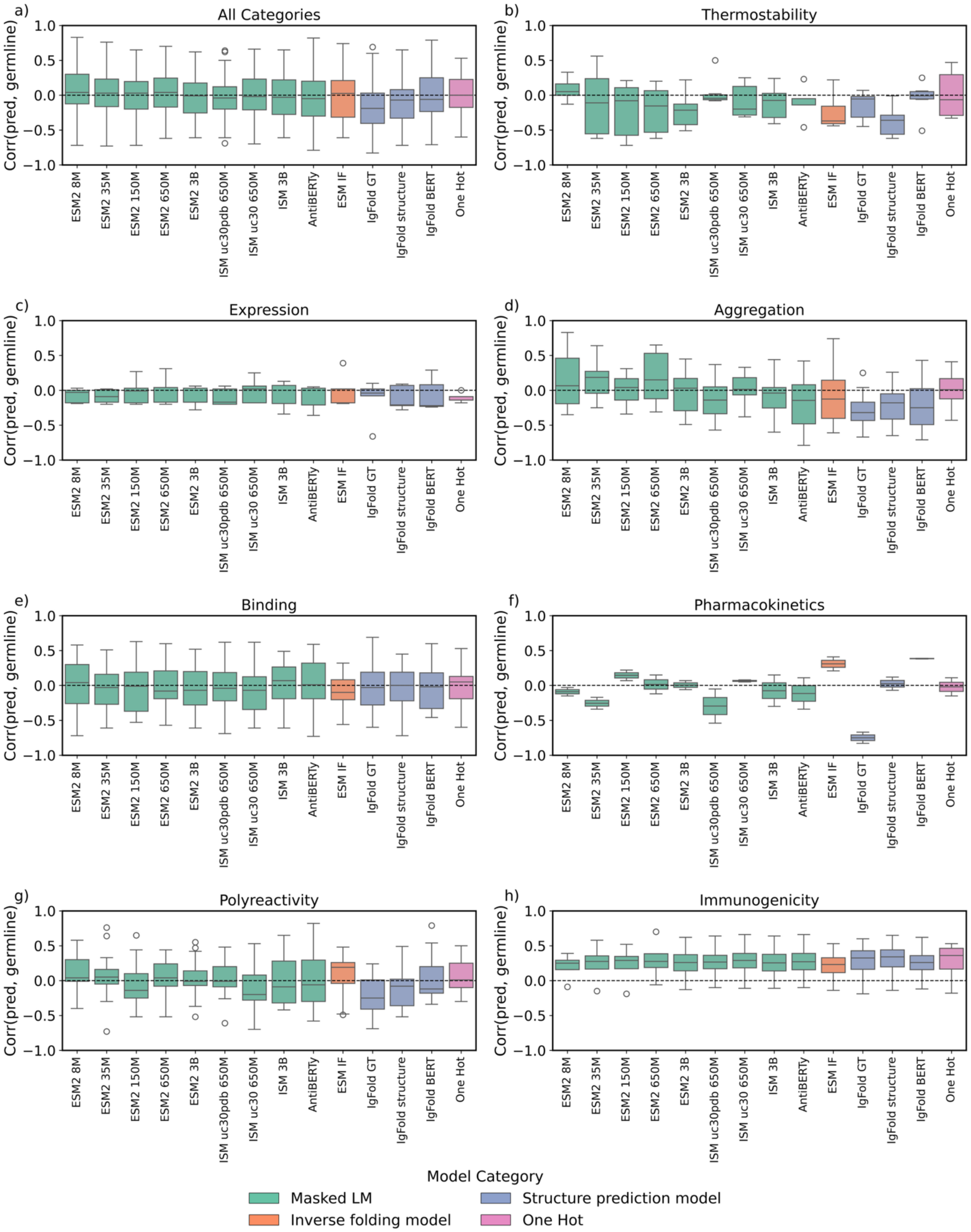

Figure S7: Distribution of few-shot prediction correlations with germline. Models are colored based on their architecture, and the *y*-axis displays the range of Spearman's correlations between predicted developability and the distance from germline. A correlation approaching 1.0 indicates the model is highly

bioRxiv preprint doi: https://doi.org/10.64898/2025.12.27.696706; this version posted December 27, 2025. The copyright holder for this preprint (which was not certified by peer review) is the author/funder, who has granted bioRxiv a license to display the preprint in perpetuity. It is made available under aCC-BY 4.0 International license.

confident in germline sequences and highly unconfident in sequences far from germline. We only report Spearman's correlations with datasets that exhibit at least five significant dataset-model p-values to ensure our conclusions are statistically sound.

Table S19: Few-shot correlation with germline signal. Tabulated correlation between model confidence and distance from germline. A correlation approaching 1.0 indicates the model is highly confident in germline sequences and highly unconfident in sequences far from germline.

Scoring Method	All Categories	Tm.	Exp.	Agg.	Bind.	PK.	Poly.	Imm.
AntiBERTy	-0.05	-0.05	-0.21	-0.15	0.01	-0.12	-0.06	0.27
ESM2 8M	0.04	**0.05**	-0.03	0.07	0.04	-0.09	0.04	0.25
ESM2 35M	0.03	-0.11	-0.09	**0.19**	-0.03	-0.26	0.05	0.28
ESM2 150M	0.03	-0.08	-0.01	0.04	-0.01	0.15	-0.14	0.29
ESM2 650M	**0.04**	-0.16	-0.17	0.15	-0.08	0.02	0.04	0.28
ESM2 3B	-0.01	-0.22	-0.17	0.03	-0.07	0.01	-0.01	0.26
ISM uc30 650M	-0.02	-0.20	**0.02**	0.02	-0.07	0.07	-0.20	0.29
ISM uc30pdb 650M	-0.04	-0.05	-0.17	-0.14	-0.04	-0.30	-0.01	0.27
ISM 3B	-0.03	-0.08	-0.19	-0.04	**0.07**	-0.08	-0.09	0.26
ESM IF	0.03	-0.37	-0.18	-0.13	-0.10	0.31	**0.19**	0.23
IgFold BERT	-0.06	-0.01	-0.22	-0.25	-0.02	**0.39**	-0.12	0.26
IgFold GT	-0.19	-0.06	-0.04	-0.32	-0.03	-0.75	-0.25	0.33
IgFold structure	-0.07	-0.36	-0.21	-0.18	0.00	0.02	-0.08	0.34
One Hot	0.00	-0.07	-0.09	0.01	0.05	-0.02	0.01	**0.36**

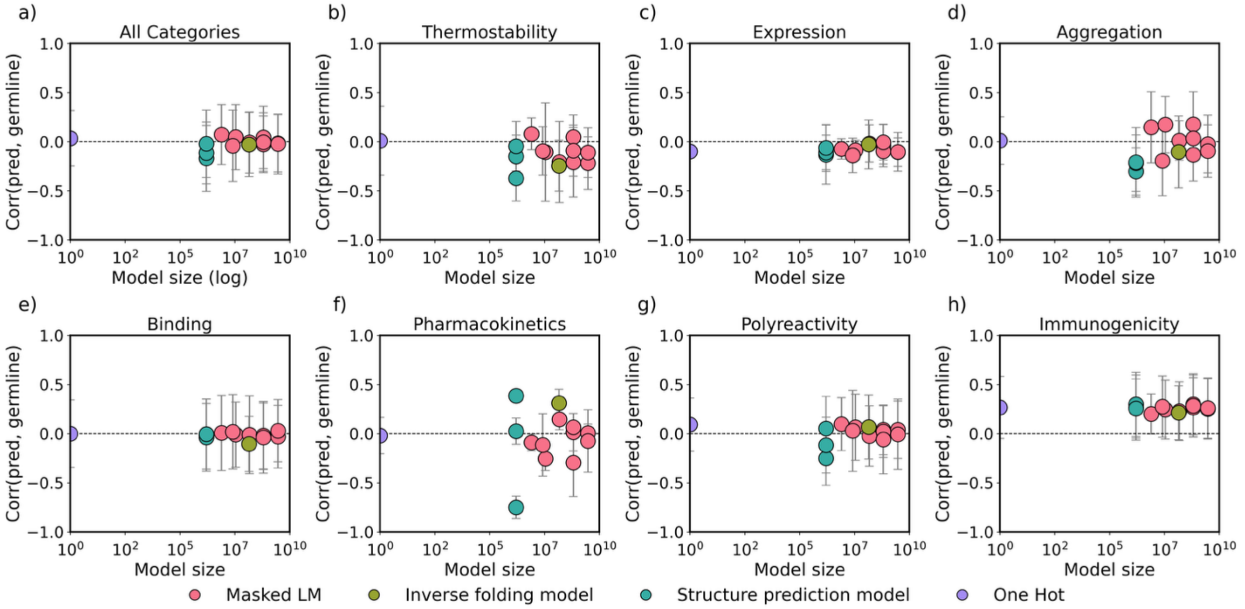

Figure S8: Distribution of few-shot prediction correlations with germline compared to parameter size. Models are colored based on their architecture, and the y-axis displays the range of Spearman's correlations between predicted fitness and the distance from germline. A correlation approaching 1.0 indicates the model is highly confident in germline sequences and highly unconfident in sequences far from

bioRxiv preprint doi: https://doi.org/10.64898/2025.12.27.696706; this version posted December 27, 2025. The copyright holder for this preprint (which was not certified by peer review) is the author/funder, who has granted bioRxiv a license to display the preprint in perpetuity. It is made available under aCC-BY 4.0 International license.

germline. We only report Spearman's correlations with datasets that exhibit at least five significant dataset-model p-values to ensure our conclusions are statistically sound.
